# Methylene blue retains in vitro activity against early ring-stage artemisinin-resistant *Plasmodium falciparum*

**DOI:** 10.1186/s12936-026-05928-7

**Published:** 2026-05-05

**Authors:** Olivia Verdier, Peter Christensen, Chalita Kaewkanya, Pachinee Kobphan, Candy Beau, Aung Pyae Phyo, Mallika Imwong, Kesinee Chotivanich, Nicholas J. White, François Nosten, Victor Chaumeau

**Affiliations:** 1 Shoklo Malaria Research Unit, Faculty of Tropical Medicine, Mahidol-Oxford Tropical Medicine Research Unit, Mahidol University, Mae Ramat, Thailand; 2 Centre for Tropical Medicine and Global Health, Nuffield Department of Medicine, University of Oxford, Oxford, UK; 3 Mahidol-Oxford Tropical Medicine Research Unit, Faculty of Tropical Medicine, Mahidol University, Bangkok, Thailand; 4 Department of Molecular Tropical Medicine and Genetics, Faculty of Tropical Medicine, Mahidol University, Bangkok, Thailand; 5 Department of Clinical Tropical Medicine, Faculty of Tropical Medicine, Mahidol University, Bangkok, Thailand

**Keywords:** Methylene blue, In vitro sensitivity, *Plasmodium falciparum*, Artemisinin resistance, *kelch13*, P441L, R561H, Ring survival assay, Thailand-Myanmar border, E_max_ model

## Abstract

Artemisinin resistance in *Plasmodium falciparum* is now well established in three continents and challenges the efficacy of antimalarial drug regimens. This study examined whether methylene blue, an ancient antimalarial drug with a broad spectrum of activity against *Plasmodium* blood stages, retains its activity against the young rings of artemisinin-resistant parasites. Clinical isolates carrying *kelch13* wild type (n = 3), R561H (n = 9), or P441L (n = 10) genotypes were tested with a modified ring survival assay whereby 0- to 3-h post-invasion rings were exposed to a range of methylene blue concentrations. Ring survival was analysed with a Bayesian mixed effects E_max_ model accounting for variability across isolates and experimental replicates. Methylene blue suppressed ring-stage survival at low nanomolar concentrations with no evidence of *kelch13*-mediated cross-resistance: the mean 50% inhibitory concentration (IC_50_) estimates were 23 nM (95% credible interval [CrI]: 15 to 37) for wild type, 27 nM (95% CrI: 21 to 36) for R561H and 14 nM (95% CrI: 10 to 20) for P441L. These findings indicate that methylene blue remains active in vitro against ring-stage artemisinin-resistant parasites.

**Supplementary Information:**

The online version contains supplementary material available at 10.1186/s12936-026-05928-7.

## Introduction

*Falciparum* malaria remains a devastating infectious disease globally, with an estimated 245 million cases and 698,000 deaths in 2022, mostly among children under five and pregnant women in sub-Saharan Africa [1].

Antimalarial drugs play a crucial role in the treatment of malaria [2] and artemisinin derivatives have become the cornerstone of antimalarial drug regimens used worldwide [3]. This is because artemisinins are potent, fast-acting, well-tolerated drugs with broad activity spectrum, including circulating young rings (thereby quickly reducing parasitaemia and preventing development of more pathological cytoadhering mature stages) [4] and immature gametocytes (thereby reducing transmission) [5]. Artesunate is the treatment of choice for severe malaria [6]. In a pivotal randomised controlled trial of parenteral artesunate *versus* quinine in African children, artesunate reduced mortality and the development of coma and convulsions, and caused less post-treatment hypoglycaemia [7].

Artemisinin resistance, characterised by slower parasite clearance in vivo, has emerged and spread independently in Southeast Asia, East Africa and South America, driven by mutations in the *kelch13* propeller domain [8]. Transcriptomic studies showed that resistance is associated with an altered gene expression profile during early asexual blood stage cycle linked with increased stress response and asexual life changes conferring young ring stages the ability to survive artemisinin exposures and resume their development after drug clearance [9]. Hence, artemisinin resistance is not detected in standard in vitro drug susceptibility tests whereby parasites are exposed to low drug concentrations for 48–72 h [10]. An alternative phenotypic ring survival assay has been developed, in which synchronised ring stages are exposed to a single high dose of drug for a short time and ring survival is assessed after one cycle of re-invasion [11]. The current ring survival assay consists of a single-concentration assessment and does not provide information on the characteristics of the dose–response. Furthermore, per isolate estimates of the survival rates are usually combined to produce summary statistics that reflect the population distribution but this approach is vulnerable to selection bias and does not accommodate intra- and inter-experiment random variability [12]. Recently, a Bayesian approach to assessment of dose–response in vitro was developed but it has not been tested for antimalarial drug testing [13].

Methylene blue, the first synthetic antimalarial, is a phenothiazine dye discovered at the end of the nineteenth century [14]. Early observation that it can stain alive malaria parasites and concentrates in parasitised red blood cells motivated its assessment in the treatment of malaria [15]. Methylene blue gradually fell out of clinical use following the introduction of synthetic quinoline antimalarials, which became widely adopted during the twentieth century. Additionally, the compound was associated with blue discoloration of urine and sclera that affected patient acceptability [16]. Historical and economic factors may also have contributed to its decline, including changes in pharmaceutical production and market dynamics for antimalarial drugs [17, 18]. Interestingly, it remained frequently used only in some countries, in particular for rescuing treatment failures with quinine [19]. Methylene blue was systematically re-evaluated only 100 years later in the context of increasing drug resistance worldwide [17]. Since its mode of action is different than that of artemisinins [8, 18], it could be useful in areas where resistance to the latter has emerged. Whether the mechanisms involved in artemisinin resistance could also compromise the antimalarial drug effects of methylene blue remains to be elucidated.

Methylene blue is active against *Plasmodium* asexual blood stages (including young rings) with 50% inhibitory concentration (IC_50_) in the nanomolar range against chloroquine and multi-drug resistant parasites, using schizont growth as an outcome [20–24]. Activity against artemisinin-resistant parasites is not well known: only the laboratory-adapted K1 clone and two field isolates were tested in vitro [25, 26]. When used alone, parasite clearance is slow and long treatments are needed to cure the infection [16]. Previous studies have reported synergistic effects of methylene blue with quinine, pyrimethamine and artemisinins, but not with chloroquine. However, the clinical relevance of these interactions remains uncertain [22, 27]. Additionally, methylene blue has strong and rapid transmission-blocking effects against the gametocytes [28] but is not active against liver stages [29].

This study sought to assess the activity of methylene blue against the ring stage of artemisinin-resistant *P. falciparum* isolates from the Thai-Myanmar border with a modified ring survival assay capturing the dose–response relationship between drug exposures and parasite survival, to test whether *kelch13* mutations confer cross-resistance to methylene blue in vitro.

## Methods

### Participants and parasite samples

A total of 28 *P. falciparum* isolates were sourced from the cryopreserved collection maintained by the Shoklo Malaria Research Unit, Mae Ramat, Thailand. These isolates originated from patients attending clinics along the Thai-Myanmar border between 2010 and 2014. They were selected based on *kelch13* genotype including wild type (n = 3), R561H (n = 9) and P441L (n = 10) because these mutations are now predominant in this area [30]. The well-characterised, culture-adapted NF54 strain was used as reference to validate the assay. Artemisinin resistance was assessed by determining parasite clearance and *kelch13* genotype as described previously [30].

### Compound

Proveblue®, a pharmaceutical-grade formulation of methylene blue that shows in vitro antiplasmodial activity against drug-resistant *Plasmodium falciparum* [24], was kindly provided by Provepharm (Marseille, France) and used as a 13,370 µM stock solution (methylene blue trihydrate 50 mg/10 mL). Single-use aliquots were made from unopened vials and stored at 4 °C protected from light until use.

### Parasite culture and synchronisation

Parasite culture and ring survival assay were performed following procedures described previously with some modifications to allow for batch testing and assessment of the dose–response [11]. Incomplete culture medium was composed of RPMI-1640 (Sigma-Aldrich, catalog no. R6504), supplemented with 2 g/L of glucose (Sigma-Aldrich, catalog no. G7528), 2 g/L of NaHCO3 (Sigma-Aldrich, catalog no. S6014), 5.7 g/L of HEPES (Sigma-Aldrich, catalog no. H4034), 18 mg/L of hypoxanthine (Sigma-Aldrich, catalog no. H9636) and 40 mg/L of gentamicin (L.B.S. Laboratory Co. Ltd., registration no. 1A 396/30). Complete culture medium consisted of incomplete medium supplemented with 10% heat-inactivated serum matched to the sample’s blood group. Cultures were maintained at 2–3% haematocrit and incubated at 37 °C in a 90% N₂, 5% CO₂, and 5% O₂ atmosphere. Parasite numbers were expanded and partially synchronised with D-sorbitol (Sigma-Aldrich, catalog no. S6021) until ring population predominated before cryopreservation in glycerolyte (Sigma-Aldrich, catalog no. G2025). Parasites were thawed, cultured to schizont stages, purified using a Percoll gradient (Merck, catalog no. P1644) and allowed to invade for 3 h on fresh red blood cells before D-sorbitol and washing treatment. The resultant 0- to 3-h post-invasion rings were used in the ring survival assay.

### Ring survival assay

0–3 h post-invasion rings were exposed to various methylene blue dilutions (0, 5.14, 10.28, 51.4, 102.8, 257, 514, 1028 nM) in triplicate on 96-well plates (Thermo Scientific, catalog no. AB2800) for 6 h. Working dilutions were prepared extemporaneously in culture medium and plates were kept shielded from light to limit photoreduction of methylene blue. Cultures were washed once to remove methylene blue and incubated in fresh medium for an additional 66 h after exposure. After 72 h total incubation, thin smears were prepared on clean glass slides (Sail Brand, catalog no. 7105), fixed in methanol (VWR International, catalog no. 20847.307), and stained for 10 min with 10% Giemsa (Merck, catalog no. 1.09204.0500). The proportion of infected red blood cells was assessed by counting the number of viable asexual parasites per 10,000 red blood cells. Pyknotic forms, vacuolated parasites, and gametocytes were excluded. To facilitate counting, the average number of red blood cells per field was estimated by counting the number of red blood cells in one representative field of observation, and the value was used to calculate the number of fields to reach at least 10,000 red blood cells. Each slide was read independently by two microscopists, and the average parasite count was used in the analysis.

### Data analysis

In the classical assessment of single-concentration ring survival assay, ring survival (defined as the ratio between the proportion of infected red blood cells in treated wells and the mean proportion of infected red blood cells in the drug-free wells) was analysed under a multilevel logistic regression model including *kelch13* genotype as a linear predictor and a random effect across tests to account for correlation in ring survival between experimental replicates within the same test. The relative risk was then calculated using the odds ratio estimate of pairwise comparisons between groups and overall survival of wild type parasites. A concentration of 50 nM was used in this assessment because it was the concentration closest to IC_50_ in dose–response analysis.

In the analysis of dose–response data, ring survival was quantified under a Bayesian E_max_ multilevel model taking into account variability across experiments and technical replicates (Additional file 1 Supplementary Methods and Figures S1-S4). NF54 and isolate data were analysed separately to account for potential differences in the characteristics of the dose–response between NF54 and wild type isolates, and repeated testing of NF54.

## Results

**Fig. 1:**
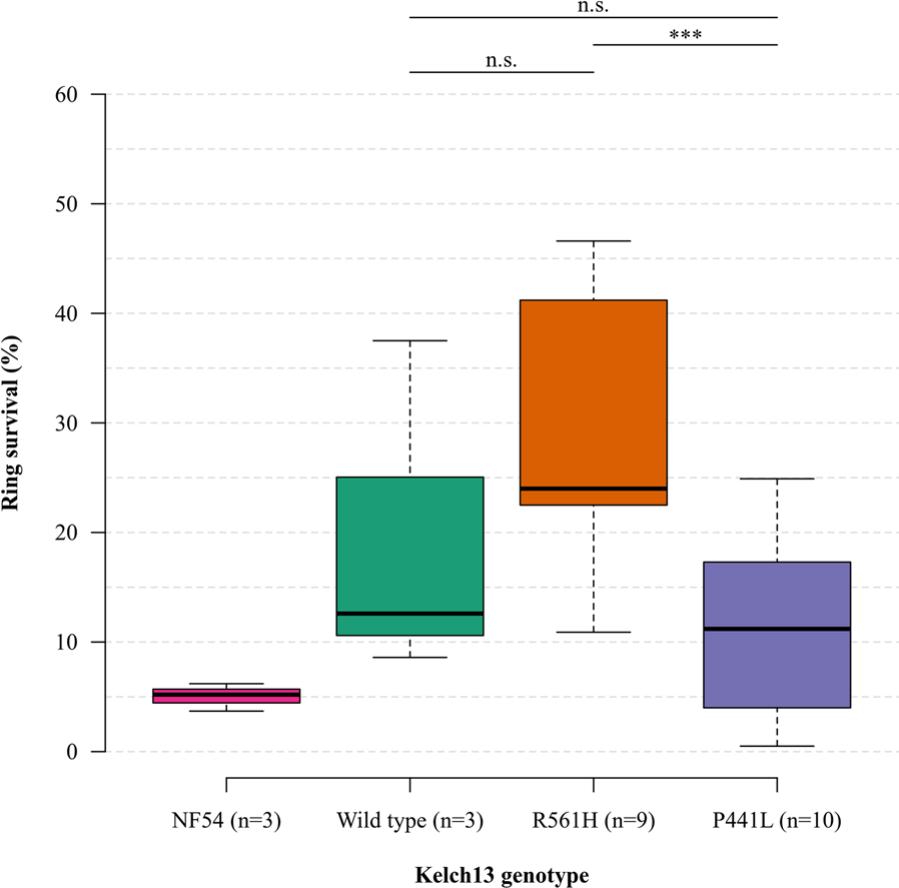
Distribution of observed ring survival in the ring survival assay at a single methylene blue concentration of 50 nM across experimental groups (NF54 reference strain and clinical isolates with different *kelch13* genotypes)

A total of twenty-five ring survival assay experiments were conducted with three wild type, nine R561H and ten P441L isolates, together with three experiment repeats carried out with NF54 (Additional file 1 Table S1). For wild type parasites, the mean survival after 50 nM exposures was 10%, 37%, and 13% (n = 3). For R561H, the median survival was 24% (IQR 22–42; range 11–46; n = 9). The corresponding figures for P441L mutants were 12% (IQR 5–17; range 0.4–25; n = 10) (Fig. 1). The logistic mixed-effects model explained most of the data variability (conditional R2 = 0.97; marginal R2 = 0.39). Post-hoc comparisons indicated that ring survival was significantly higher in R561H than in P441L mutants (relative risk [RR] 1.99, 95% CI 1.42–2.57), but not significantly different between wild type and either mutant group (wild type vs R561H RR 0.66, 95% CI 0.32–1.26; wild type vs P441L RR 1.49, 95% CI 0.81–2.32). Survival in the assay did not correlate with growth rate (Spearman correlation coefficient = − 0.033, p-value = 0.8728). For NF54, the observed mean survival across three independent experiments was 5% (SD 1.3%), and the model estimate was 4.7% (95% CI 3.7–6.0).

**Fig. 2:**
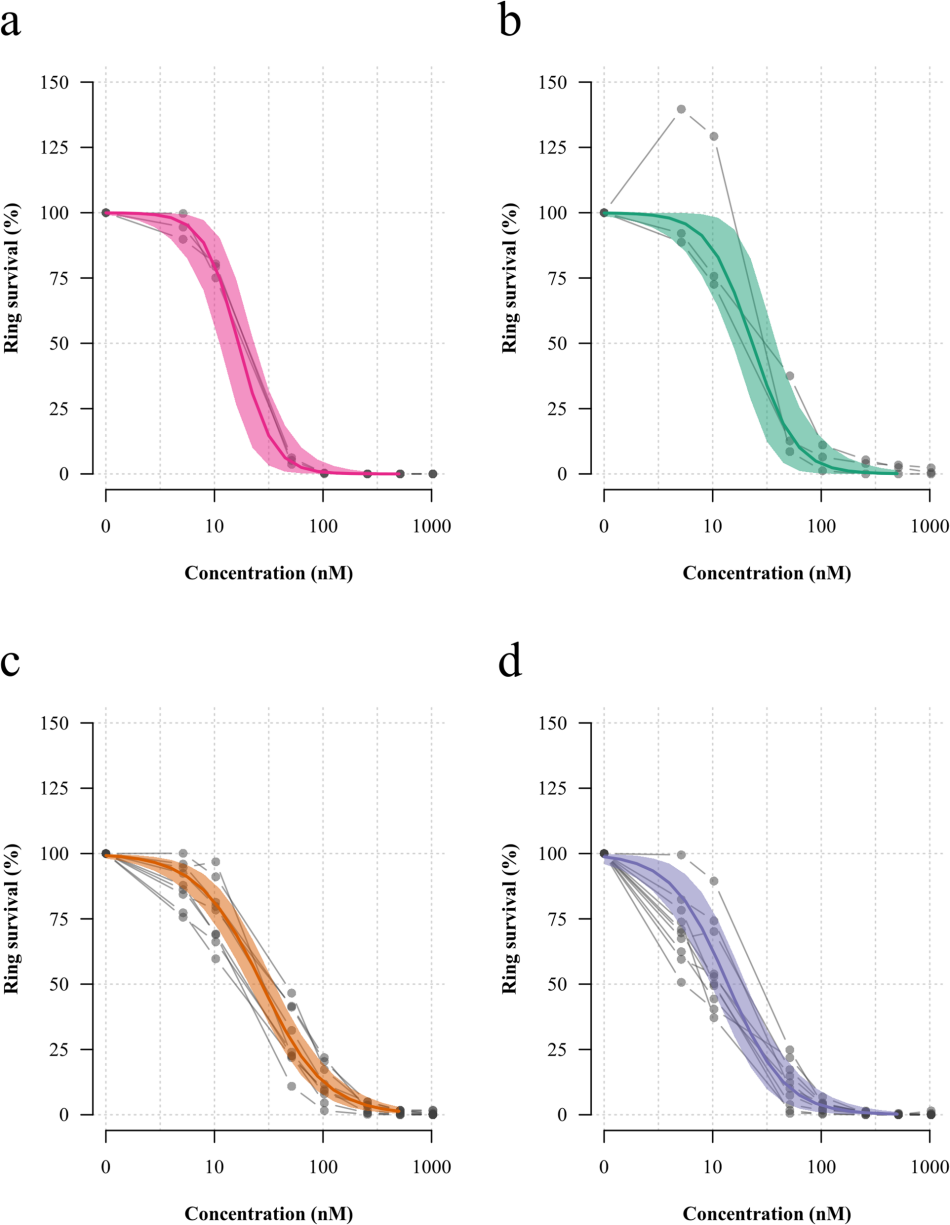
Concentration–response relationship between methylene blue concentration and ring survival in the ring survival assay. **A** NF54 reference strain; **B** wild type isolates, **C** R561H isolates and **D** P441L isolates. The grey dots show the observed values and the coloured-line and shaded area show the model-fitted relationship plotted using μ_IC50_population_, μ_H_population_, β_IC_50G[i]_ and β_H_G[i]_ estimates given by the model output and the corresponding 95% credible interval, respectively

Parameter estimates for the Bayesian multilevel E_max_ models are presented in Table 1. In the isolate dataset, the model estimated a population mean IC_50_ of 23 nM (95% CrI 15–37) and a Hill coefficient of 2.2 (1.4–4.1). Methylene blue maintained low-nanomolar activity across all *kelch13* genotypes (Table 2). R561H isolates were slightly less susceptible than wild type, whereas P441L mutants were marginally more sensitive. These differences were small, and only the contrast between R561H and P441L reached statistical credibility (Additional file 1 Table S2). The NF54 reference strain showed comparable parameters (IC_50_ 17 nM, 95% CrI 11–23; H 2.7 [1.8–4.1]). The model described the data well (Fig. 2), and assay variability was small (Additional file 1 Figure S5), with IC_50_ and H values varying by less than one-fold across isolates and replicates.

**Table 1:** Parameter estimates given by the output of Bayesian multilevel Emax models using in the analysis of clinical isolate and NF54 data

Parameter	Clinical isolates		NF54	
	Median of posterior draws	95% CrI	Median of posterior draws	95% CrI
μ_E0_population_	1.16	0.81–1.52	6.28	4.08–8.36
σ_E0_test_	0.65	0.42–1.14	0.16	0.05–0.98
μ_IC50_population_	23.2	14.8–37.2	16.6	10.8–22.7
σ_IC50_test_	0.33	0.19–0.57	0.12	0.01–0.98
σ_IC50_replicate_	0.14	0.08–0.23	0.12	0.04–0.28
μ_H_population_	2.19	1.41–4.06	2.75	1.78–4.09
σ_H_test_	0.2	0.06–0.4	0.13	0.01–1.06
σ_H_replicate_	0.15	0.08–0.25	0.09	0.01–0.33
β_ IC_50 wild-type_	1	Reference	–	–
β_ IC_50 R561H_	1.17	0.69–1.92	–	–
β_ IC_50 P441L_	0.6	0.34–1.06	–	–
β_H_wild-type_	1	Reference	–	–
β_H_R561H_	0.67	0.36–1.1	–	–
β_H_P441L_	0.76	0.39–1.31	–	–
σ_residual_	0.14	0.13–0.15	0.18	0.15–0.22

**Table 2:** Characteristics of the dose–response relationship between exposure to methylene blue and ring survival in the ring survival assay for clinical isolates and NF54

Experimental group	IC_50_ (nM)^a^	H^a^	ED90 (nM)^a^	ED99 (nM)^a^
Wild type isolates	23 (15–37)	2.2 (1.4–4.1)	64 (34–124)	190 (68–625)
R561H isolates	27 (21–36)	1.5 (1.2–1.8)	121 (82–179)	610 (320–1230)
P441L isolates	14 (10–20)	1.7 (1.2–2.3)	52 (31–89)	218 (94–565)
NF54	17 (11–23)	2.7 (1.8–4.1)	37 (22–63)	88 (44–221)

^a^ Values are median of posterior draws and 95% credible interval

## Discussion

This study demonstrates that Proveblue® (the only Good Manufacturing Practices compliant methylene blue formulation currently on the market) exhibits in vitro activity against both artemisinin-sensitive and resistant *P. falciparum* isolates from the Thai-Myanmar border, with IC_50_ values in the nanomolar range using ring survival as the outcome. Although differences in susceptibility were detected between *kelch13* genotypes (R561H mutants showing slightly higher IC_50_ and ED_90_ values than P441L mutants), the magnitude of these differences was small and of uncertain biological significance. These findings support the hypothesis that methylene blue retains efficacy against ring-stage artemisinin-resistant parasites and could be considered as a complementary or partner component in artemisinin therapies. Although methylene blue should not be used by individuals with glucose-6-phosphate dehydrogenase deficiency and during pregnancy because of the risk of haemolytic anaemia, it is already licensed for the treatment of methaemoglobinaemia and used in other medical contexts (surgical staining, oncology), facilitating potential repurposing as an antimalarial [17].

Use of methylene blue for malaria chemotherapy requires careful consideration of its pharmacodynamic and pharmacokinetic properties. Methylene blue has a t_max_ of ~ 2 h after oral administration and a half-life of ~ 6 h comparable to that of artemisinins. Similar dosing gives higher C_max_ with parenteral than oral formulation [31]. In vitro studies have also reported synergistic interactions between methylene blue and artemisinin derivatives but the precise mechanisms involved and clinical relevance of this observation remain uncertain [22]. Methylene blue is slow-acting in vivo and seven-day treatments (4 asexual cycles) are needed to kill all parasites [16]. Therefore, it is probably not valuable as an adjuvant to artemisinin-combination therapies used for the oral treatment of uncomplicated malaria. This statement is further supported by the similar parasite clearance and cure rate when comparing artesunate-amodiaquine *versus* artesunate-amodiaquine-methylene blue [32]. However, parenteral methylene blue combined with artesunate may be useful in the treatment of severe malaria in areas where artemisinin-resistance is prevalent. Activity of methylene blue against artemisinin-resistant ring stages suggests that it could help preserve the therapeutic advantage of artesunate over quinine in resistant infections, although this requires confirmation in vivo. Methylene blue effects on cytoadherence and rosetting have not been assessed but observations from recent primate models suggest this mechanism could also contribute [33].

The absence of cross-resistance between artemisinin and methylene blue may reflect differences in their antimalarial mechanisms, including the redox-targeting effects of methylene blue [21, 34]. The IC_50_ values estimated in this modified ring survival assay are higher than those reported in previous studies using standard in vitro and ex vivo assays. The higher IC_50_ estimates observed here are most likely explained by assay design rather than by reduced intrinsic susceptibility. To date, there is no clear methylene blue resistance in *P. falciparum*, and the potential for selecting de novo resistance mutations under conditions of sublethal exposures is deemed low [35].

A strength of this study is the use of a modified ring survival assay combined with Bayesian multilevel modelling, which enabled estimation of the dose–response relationship while accounting for variability between isolates and experimental replicates. This approach reduces sensitivity to outliers and improves power to detect genotype-associated differences in parasite susceptibility [12]. Repeated testing of the reference strain NF54 also provides a benchmark for intra- and inter-experiment variability. The large variability for clinical isolates may be due to unknown *kelch13*-independent biological factors. This requires further investigation. Survival in the assay did not correlate with growth rate but the lower parasite densities may explain the larger variability in the isolate dataset.

Certain limitations should be acknowledged. The sample size was small and only two *kelch13* mutations were assessed (R561H and P441L). These alleles were chosen because they are now predominant in Karen state (Eastern Myanmar) [30]. It was not possible to include additional mutations in this assessment because testing capacity was limited, and the modified assay is resource-intensive to implement. Given the differences in survival between groups, it is important that other alleles, such as C580Y and F446I, are investigated to confirm the applicability of the results to other genotypes before methylene blue-artesunate combination trials in severe *falciparum* malaria in East Africa are contemplated. In vitro effects on parasite growth do not necessarily correlate with parasite clearance in vivo, especially considering the central role of spleen in removing damaged parasites and red blood cells from the circulation after drug exposures [36]. The culture history of parasite isolates used in this study including passage number is not known. Additionally, culture adaptation was associated with increased drug susceptibility in a study in Cambodia but the magnitude of the change was small and assay variability was not taken into account [37].

Future randomised trials should evaluate whether short course parenteral methylene blue added to standard intravenous artesunate can safely enhance parasite clearance and reduce mortality in severe *falciparum* malaria. Such trials would be particularly relevant in areas of East Africa where malaria incidence and mortality are high, and artemisinin resistance is firmly established.

## Conclusions

Methylene blue is active against artemisinin-resistant *P. falciparum* isolates and shows no evidence of *kelch13-*mediated cross-resistance in the tested isolates. While its pharmacodynamic profile makes it unsuitable for the oral treatment of uncomplicated malaria, the compound may have value as an intravenous adjunct in the treatment of severe infection in settings where artemisinin resistance reduces the efficacy of standard therapies.

## Supplementary Information

Additional file 1.

## Data Availability

All analysis code and data are available via an accompanying GitHub repository: [https://github.com/victorSMRU/methylene-blue-rsa] (https:/github.com/victorSMRU/methylene-blue-rsa).

## Abbreviations

CI: Confidence interval
CrI: Credible interval
IC: Inhibitory concentration
IQR: Interquartile range
RR: Relative risk
SD: Standard deviation
