# Supplementary material for: Methylene blue retains in vitro activity against early ring-stage artemisinin-resistant Plasmodium falciparum
Source: Malar J. 2026 May 5;25:249. doi: 10.1186/s12936-026-05928-7 (PMC13317430; doi:10.1186/s12936-026-05928-7)
Supplement: Supplementary file 1 — Additional file 1. [file 12936_2026_5928_MOESM1_ESM.docx]

**Supplementary methods**

Under the model, the likelihood function for the proportion of infected red blood cells (P) was a function of the drug concentration (C), proportion of infected red blood cells in the drug-free control wells (E_0_), concentration that halves ring survival (IC_50_) and Hill’s coefficient or shape parameter (H):

P = E_0_ – C^H x E_0_ / (C^H + IC_50_^H)

Isolate data were analysed with a two-level random error structure accounting for variability across tests performed with different isolates and across experimental replicates within the same test, and a fixed effect of *kelch13* genotype parameterised as a proportional variation of population mean IC_50_ and H. The model used for the analysis of NF54 data was parameterised similarly, except genotype effects on IC_50_ and H were not included, and the first level random effects were for variability across independent repeats of the assay with the same parasite rather than variability across tests performed with different isolates.

Thus, for each isolate (test) i and technical replicate k, the model parameters E_0i_, IC_50i,k_ and H_i,k_ were expressed as the product of population-level mean (μ_E0_population,_ μ_IC50_population,_ μ_H_population_), isolate-dependent random effect (ʎ_E0[i],_ ʎ_IC50[i]_ and ʎ_H[i]_) and replicate random effect (ʎ_IC50[k]_ and ʎ_H[k]_). The Student-t distribution with 7 degrees of freedom was chosen to accommodate the observed heterogeneity across isolates and replicates: ʎ_E0[i]_ ~ Student-t(7, 1, σ_E0_test_), ʎ_IC50[i]_ ~ Student-t(7, 1, σ_IC50_test_), ʎ_H[i]_ ~ Student-t(7, 1, σ_H_test_), ʎ_IC50[k]_ ~ Student-t(7, 1, σ_IC50_replicate_) and ʎ_H[k]_ ~ Student-t(7, 1, σ_H_replicate_). For a given isolate, the genotype effect β_G[i]_ was parameterised as a proportional fold-variation in μ_IC50_population_ and μ_H_population_ (β_IC_50G[i]_ and β_H_G[i]_, respectively). Thus, the likelihood of the proportion of infected red blood cells P_i,k,G[i]_ (isolate i, technical replicate k, genotype G[i]) given the parameters is:

P_i,k,G[i]_ ~ E_0i,k,G[i]_ – C^H_i,k,G[i]_ * E_0i,k,G[i]_ / (C^H_i,k,G[i]_ + IC_50i,k,G[i]_) + ε_i,k_

With:

E_0i_ = μ_E0_population_ * ʎ_E0[i]_

IC_50i,k,G[i]_ = μ_IC50_population_ * ʎ_IC50[i]_ * ʎ _IC50[k]_ * β_IC50_G[i]_

H_i,k,G[i]_ = μ_H_population_ * ʎ_H[i]_ * ʎ_H[k]_ * β_H_G[i]_

and residual error term ε_i,k_ ~ Normal(0,1).

We used weakly informative priors to help computational convergence. The models were run with 4 independent chains each consisting of 10000 iterations. Convergence of the chains was assessed by examining the values of effective sample size and Rhat and the traceplots (see Figures S1-S4 below).


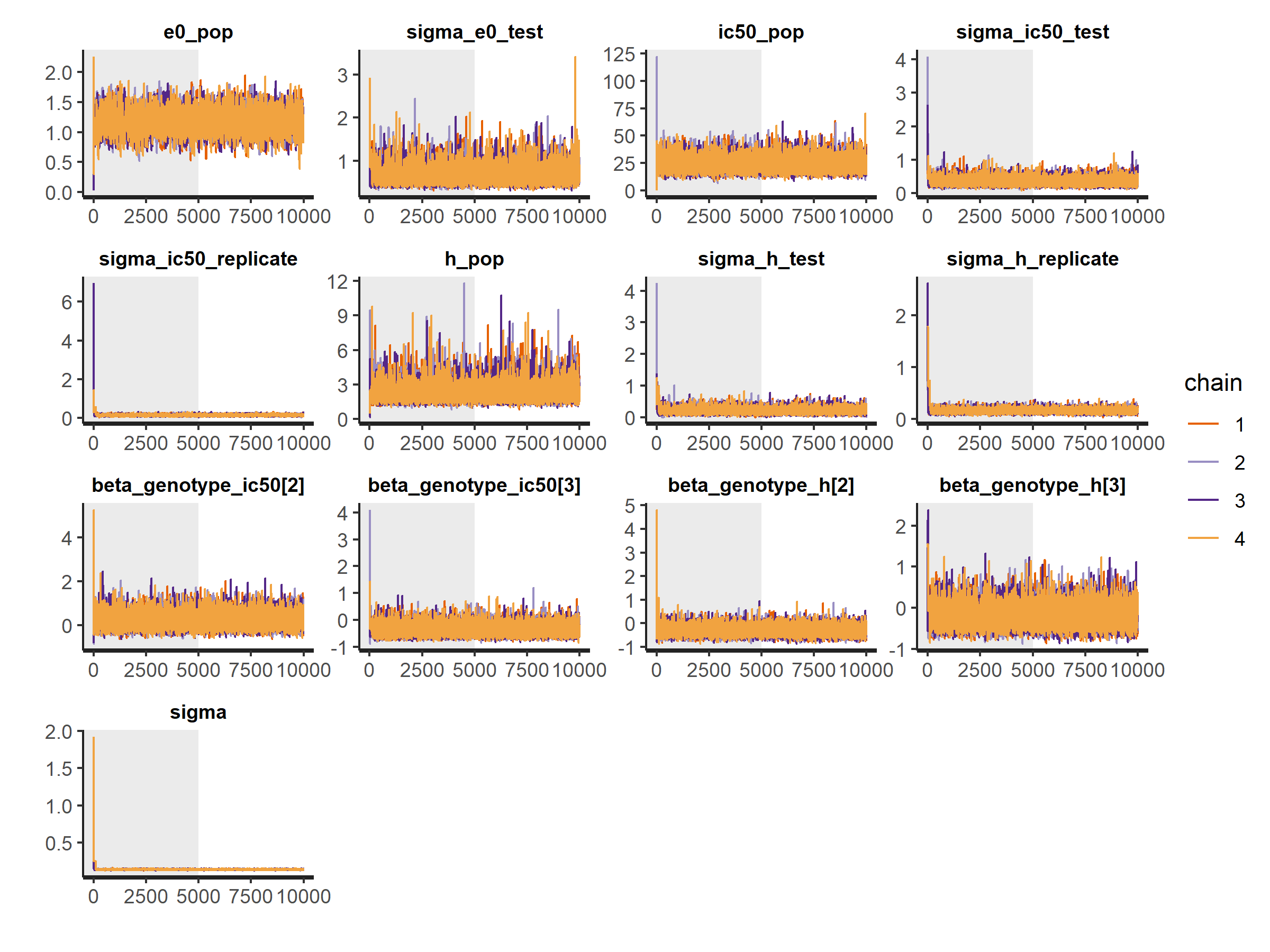


**Figure S1. Traceplot of MCMC chains showing adequate mixing and convergence for the Bayesian multilevel model fitted to clinical isolate data.**


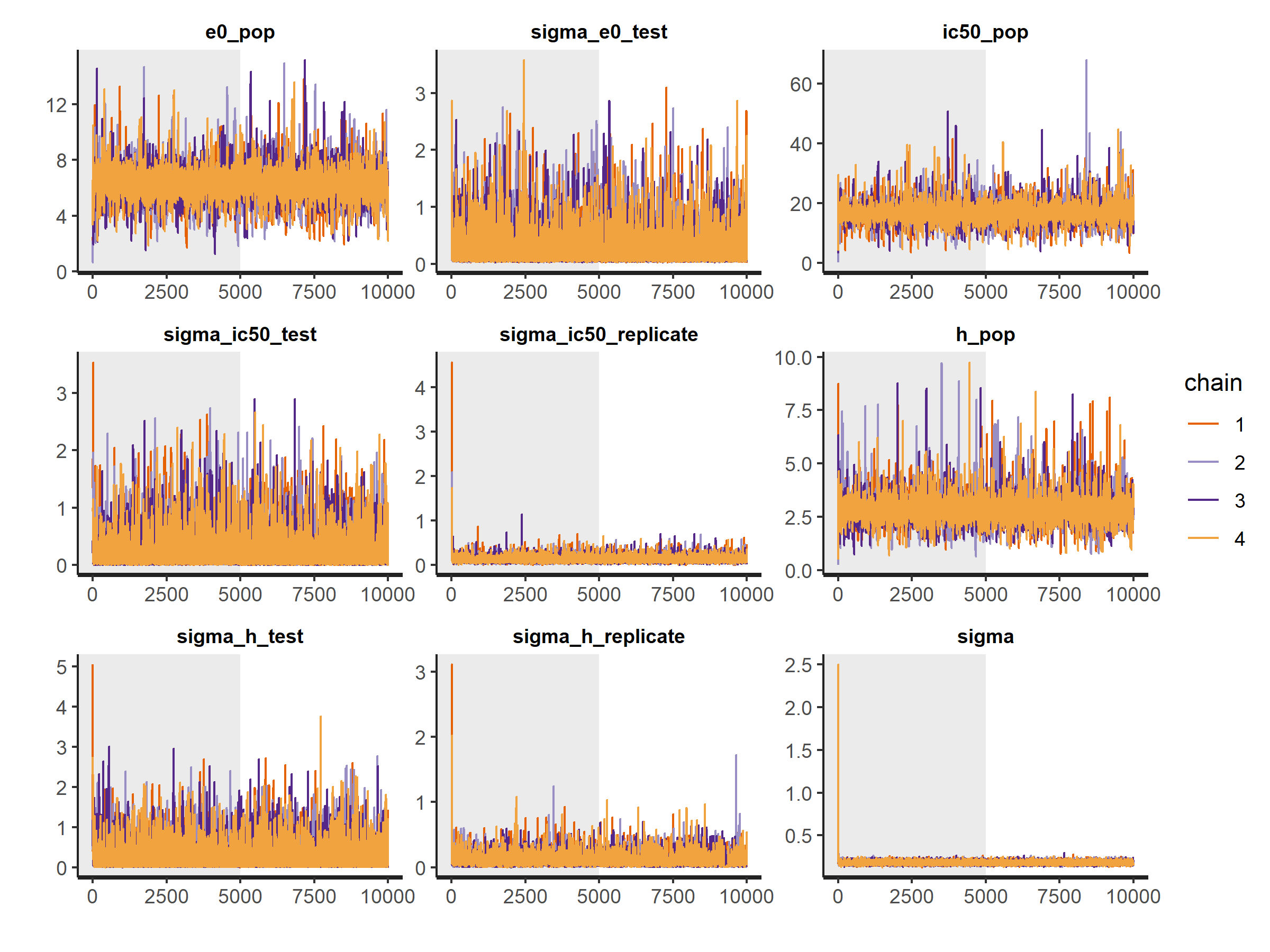


**Figure S2. Traceplot of MCMC chains showing mixing and convergence for the Bayesian multilevel model fitted to NF54 data.**


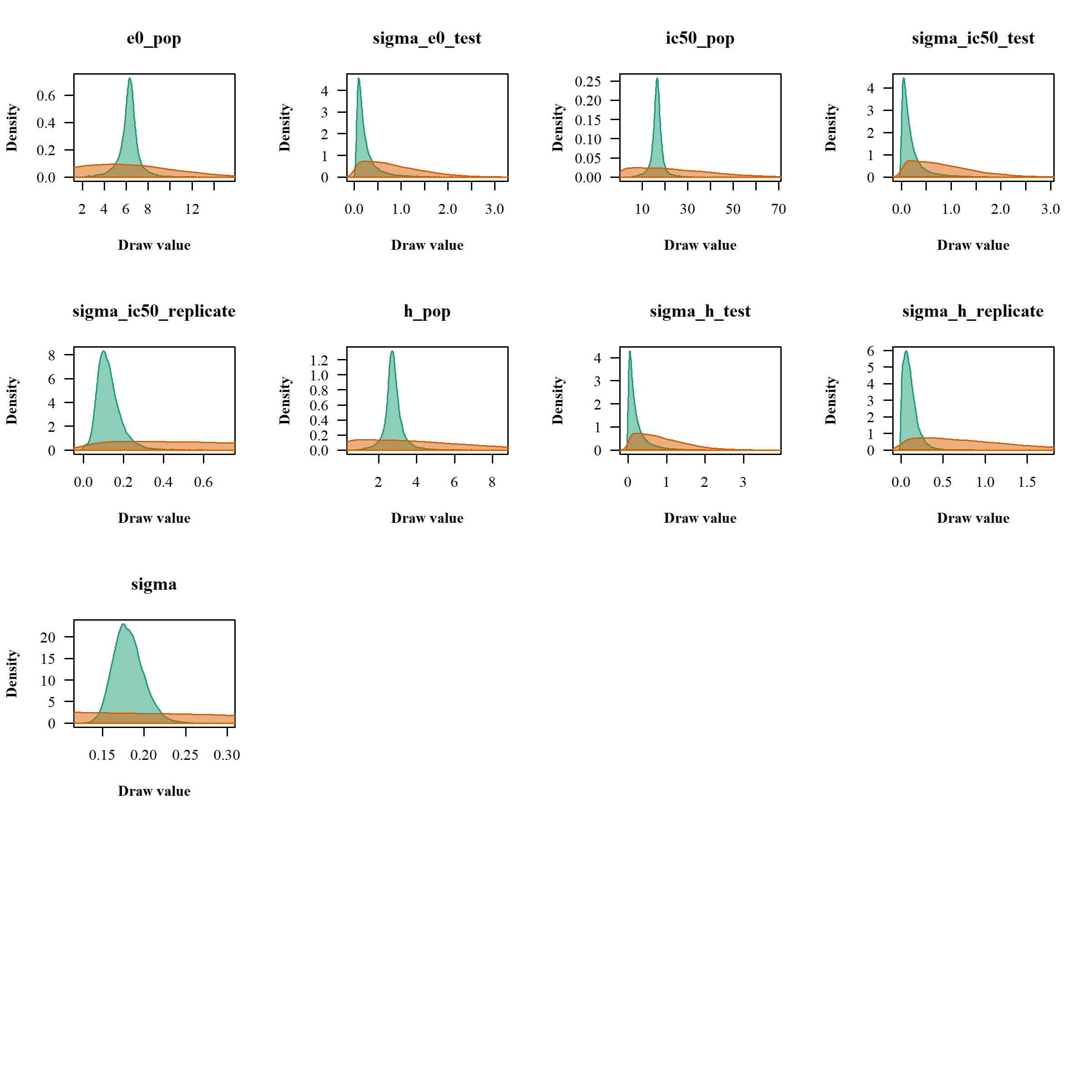


**Figure S3. Comparison between prior distributions (orange) and posterior distributions (green) for the parameters of the Bayesian multilevel model fitted to NF54 data.**


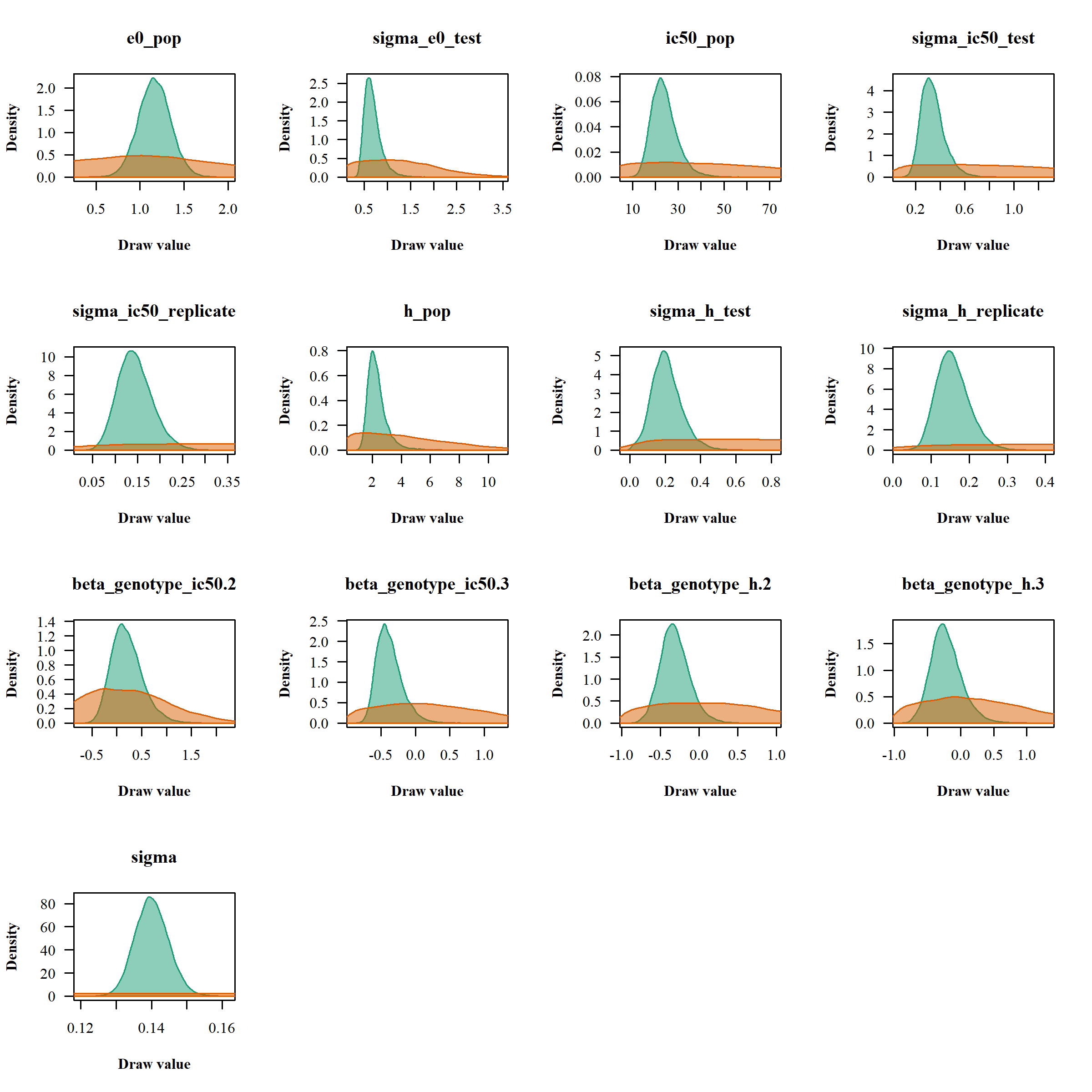


**Figure S4. Comparison between prior distributions (orange) and posterior distributions (green) for the parameters of the Bayesian multilevel model fitted to clinical isolate data.**


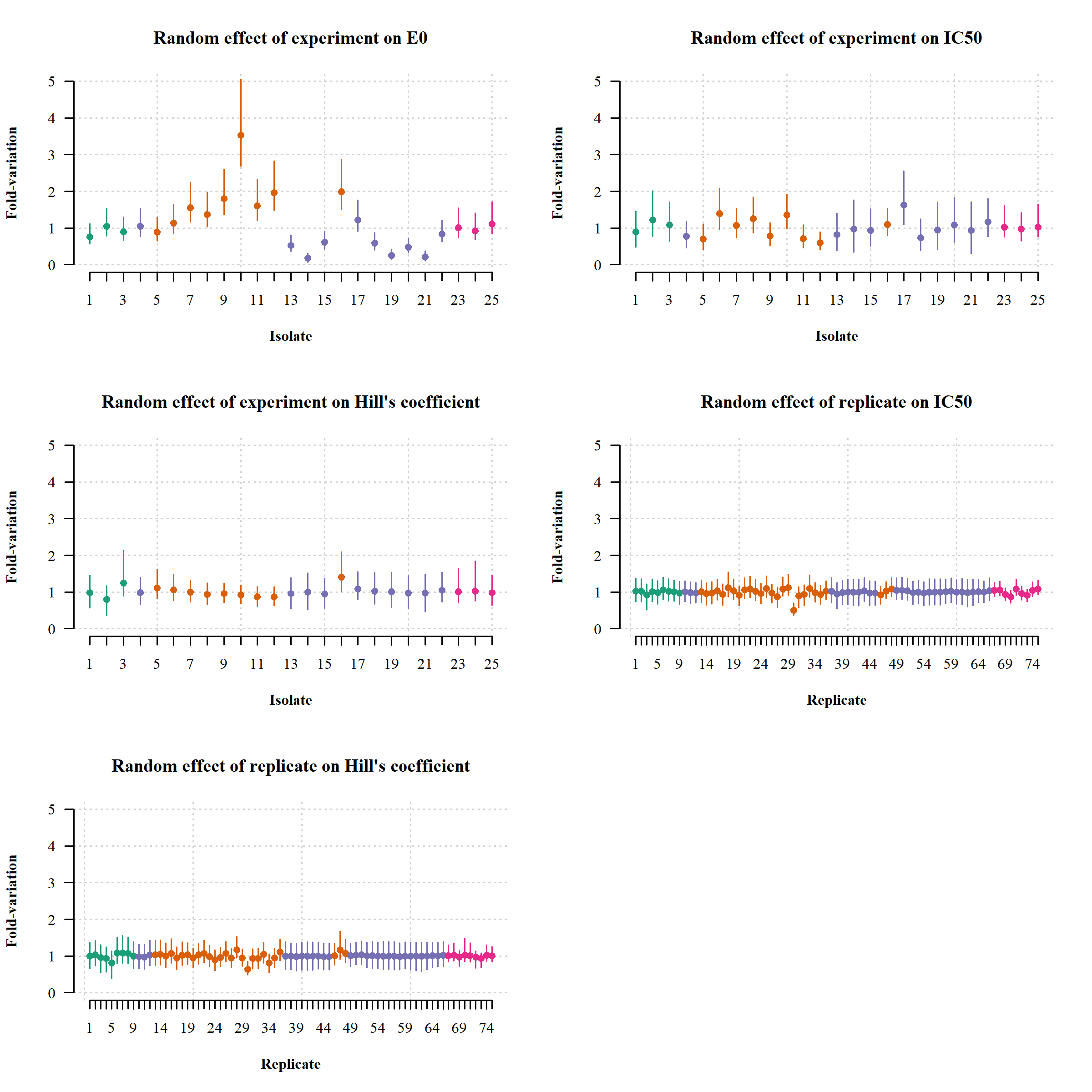


**Figure S5. Random effects of experiment and replicate on E_max_ model parameters E_0_, IC_50_ and Hill’s coefficient.** Green: wild-type, orange: R561H, purple: P441L and pink: NF54.

Table S1. Summary results of the ring survival assay experiments conducted in this study.

| **Test no.** | **Isolate / Strain** | ***Kelch13*** | **Growth rate** | **Mean ring survival at 50 nM (SD)** | **IC_50_** **nM**  **(95% CrI)** | **IC90 nM**  **(95% CrI)** | **IC99 nM**  **(95% CrI)** | **H**  **(95% CrI)** |
| --- | --- | --- | --- | --- | --- | --- | --- | --- |
| 1 | Isolate #1 | Wild type | 2.0 | 13 (3.6) | 20 (12 - 34) | 59 (28 - 122) | 183 (57 - 706) | 2.1 (1.3 - 4.07) |
| 2 | Isolate #2 | Wild type | 1.8 | 37 (6.6) | 29 (19 - 45) | 109 (61 - 217) | 458 (151 - 1950) | 1.66 (1.06 - 3.01) |
| 3 | Isolate #3 | Wild type | 1.6 | 10 (5.7) | 25 (18 - 35) | 55 (32 - 91) | 130 (55 - 320) | 2.79 (1.82 - 5.34) |
| 4 | Isolate #4 | P441L | 3.5 | 4 (1.6) | 11 (7 - 15) | 40 (23 - 79) | 174 (66 - 607) | 1.64 (1.14 - 2.42) |
| 5 | Isolate #5 | R561H | 1.1 | 11 (4.0) | 19 (12 - 29) | 72 (39 - 131) | 311 (116 - 867) | 1.64 (1.21 - 2.43) |
| 6 | Isolate #6 | R561H | 1.7 | 46 (9.0) | 38 (27 - 53) | 155 (101 - 253) | 720 (321 - 1904) | 1.56 (1.16 - 2.22) |
| 7 | Isolate #7 | R561H | 7.5 | 32 (5.1) | 29 (22 - 39) | 131 (87 - 206) | 664 (321 - 1638) | 1.47 (1.13 - 1.95) |
| 8 | Isolate #8 | R561H | 4.1 | 42 (8.6) | 34 (25 - 47) | 169 (110 - 286) | 958 (431 - 2744) | 1.38 (1.04 - 1.84) |
| 9 | Isolate #9 | R561H | 7.5 | 22 (8.8) | 21 (16 - 29) | 101 (65 - 164) | 549 (262 - 1346) | 1.42 (1.11 - 1.81) |
| 10 | Isolate #10 | R561H | 3.4 | 46 (20.0) | 37 (28 - 47) | 187 (123 - 302) | 1086 (516 - 2822) | 1.36 (1.06 - 1.73) |
| 11 | Isolate #11 | R561H | 6.9 | 24 (6.1) | 19 (14 - 28) | 109 (66 - 196) | 716 (299 - 2211) | 1.28 (0.97 - 1.67) |
| 12 | Isolate #12 | R561H | 2.5 | 22 (8.7) | 16 (12 - 22) | 90 (55 - 156) | 580 (239 - 1637) | 1.29 (1 - 1.68) |
| 13 | Isolate #13 | P441L | 3.8 | 13 (8.7) | 11 (6 - 20) | 45 (20 - 133) | 207 (57 - 1623) | 1.58 (0.92 - 2.56) |
| 14 | Isolate #14 | P441L | 1.9 | 8 (4.1) | 13 (5 - 27) | 51 (16 - 208) | 212 (47 - 3507) | 1.66 (0.83 - 2.84) |
| 15 | Isolate #15 | P441L | 3.1 | 15 (4.5) | 13 (8 - 22) | 53 (25 - 138) | 242 (70 - 1540) | 1.57 (0.96 - 2.48) |
| 16 | Isolate #16 | R561H | 2.0 | 23 (6.3) | 30 (23 - 38) | 86 (62 - 129) | 270 (145 - 613) | 2.09 (1.51 - 2.99) |
| 17 | Isolate #17 | P441L | 3.7 | 25 (3.8) | 23 (16 - 32) | 76 (48 - 121) | 281 (128 - 662) | 1.83 (1.35 - 2.65) |
| 18 | Isolate #18 | P441L | 4.9 | 2 (0.9) | 10 (6 - 18) | 36 (18 - 90) | 147 (45 - 734) | 1.72 (1.09 - 2.76) |
| 19 | Isolate #19 | P441L | 2.3 | 0 (0.7) | 13 (6 - 25) | 49 (19 - 157) | 202 (52 - 1850) | 1.68 (0.94 - 2.84) |
| 20 | Isolate #20 | P441L | 2.4 | 18 (1.7) | 15 (8 - 27) | 61 (29 - 166) | 270 (79 - 2037) | 1.6 (0.93 - 2.63) |
| 21 | Isolate #21 | P441L | 3.7 | 22 (13.0) | 13 (4 - 26) | 51 (15 - 249) | 221 (44 - 5238) | 1.61 (0.76 - 2.75) |
| 22 | Isolate #22 | P441L | 3.0 | 10 (4.3) | 16 (11 - 24) | 58 (32 - 108) | 226 (83 - 718) | 1.75 (1.21 - 2.74) |
| 23 | NF54 | Wild type | 5.4 | 4 (1.1) | 17 (15 - 20) | 38 (29 - 48) | 88 (57 - 128) | 2.81 (2.35 - 3.65) |
| 24 | NF54 | Wild type | 6.3 | 5 (1.9) | 16 (13 - 19) | 34 (22 - 44) | 79 (40 - 120) | 2.85 (2.34 - 4.34) |
| 25 | NF54 | Wild type | 6.7 | 6 (2.1) | 17 (15 - 20) | 39 (31 - 51) | 98 (65 - 146) | 2.65 (2.2 - 3.28) |

Table S2. Pairwise differences in parameter estimates across *kelch13* genotypes in the Bayesian multilevel E_max_ model fitted to isolate data.

|  | **Median** | **95% CrI** |
| --- | --- | --- |
| **IC_50_ (nM)** |  |  |
| R561H *vs* wild type | 4 | -11 - 15 |
| P441L *vs* wild type | -9 | -24 - 1 |
| R561H *vs* P441L | 13 | 4 - 23 |
| **H** |  |  |
| R561H *vs* wild type | -0.7 | -2.6 - 0.1 |
| P441L *vs* wild type | -0.5 | -2.4 - 0.5 |
| R561H *vs* P441L | -0.2 | -0.9 - 0.4 |
| **ED90 (nM)** |  |  |
| R561H *vs* wild type | 55 | -12 - 121 |
| P441L *vs* wild type | -12 | -74 - 35 |
| R561H *vs* P441L | 68 | 15 - 130 |
| **ED99 (nM)** |  |  |
| R561H *vs* wild type | 402 | -79 - 1029 |
| P441L *vs* wild type | 24 | -406 - 385 |
| R561H *vs* P441L | 382 | -56 - 1008 |
